# Supplementary material for: The molecular aetiology of tRNA synthetase depletion: induction of a GCN4 amino acid starvation response despite homeostatic maintenance of charged tRNA levels
Source: Nucleic Acids Res. 2020 Feb 4;48(6):3071–88. doi: 10.1093/nar/gkaa055 (PMC7102972; doi:10.1093/nar/gkaa055)
Supplement: gkaa055_Supplemental_File [file gkaa055_supplemental_file.pdf]

Supplementary Materials

Stochastic simulations

Global translation model:

We use the Gillespie algorithm (1) to generate reactions taking place at random times according to their propensities. The Global Translation Model (GTM) is based on the Totally Asymmetric Simple Exclusion Process (TASEP) and has four main types of reactions: A ribosomal particle may i) enter the lattice, ii) translocate on the lattice, which uses up one tRNA, iii) leave the lattice by dropping off at any site of the lattice or terminating at the last lattice site, or iv) an uncharged tRNA may be recharged. Note that the ribosomal particles have a footprint of size W codons. In the following, 'ribosome' means the translational site of the ribosome, i.e., the translocation from the initiation lattice site $i = 0$ to $i = 1$ is only possible if the lattice site $i = W + 1$ is empty.

We start at time $t = 0$ with M empty mRNAs, each of which has N_m lattice sites. Moreover, all tRNAs are charged, $T_i^t = T_i^c$. After a dwell time τ, which is drawn from an exponential distribution determined by the propensities at that time point, the next reaction is randomly chosen.

Then, all propensities, numbers of species and state of the lattices are updated, and the procedure is repeated until the predefined end time t_{max}. We allow a certain transient time before we store the information of density profiles and the properties, in order to make sure that we reach the steady state, i.e., the total integration time considered to compute average quantities is $t = t_{max} - t_{trans}$.

Specification of parameters; Global Translation Model

A real *S. cerevisiae* cell has 10^7 codons (derived using a knowledge of mRNA numbers and types per cell (2)), 3×10^6 tRNAs (3) and 2×10^5 ribosomes (4).

We chose a downscaling factor of 20 for our reduced system, corresponding to 5×10^5 codons and 10^5 tRNAs. We include 1.5×10^3 individual mRNAs with individual length according to the gene ontology classification, see Table S11. All other parameters are downscaled accordingly: the downscaled system contains 10^4 ribosomes, 1.5×10^5 tRNAs (downscaled from total tRNAs/cell estimated at 3×10^6 (3)). The abundance of each tRNA species is assumed to be proportional to the tRNA gene copy number, with a factor of proportionality of $T_t = 526$ for the 20-fold downscaled cell (see Table S3; (57)).

Note that the number of ribosomes in the reservoir changes in time. The fraction of ribosomes in the cell which are engaged in translation has been calculated for our setup to 75%, which is close to the value reported in (6). Then, the number of free ribosomes in the cytoplasm is given by 25%, i.e.,

in our downscaled cell, we have on average 2500 ribosomes available for initiation in steady state. Note that the simulation starts with empty mRNAs, i.e., initially, all ribosomes are free.

The initial initiation rates are listed in Table S2 for the downscaled cell, with the average initiation rate amounting to $\langle\alpha\rangle = 0.15/s$.

The maximum charging rate for the tRNA-synthetases $V_{max,i}$ is estimated by dividing the total number of each amino acids incorporated into proteins during the cell lifetime by the abundance of the corresponding tRNA-synthetases (7)(8). Interestingly, the $V_{max,i}$ values highly correlate with the tRNA gene copy number. The values of the Michaelis-Menten constants $K_{M,i}$ are not known for all synthetases, but the fact that the charging level of tRNAs under physiological conditions has been estimated to be 80% across different tRNA species (9-13) implies that the ratio $\xi = V_{max,i}/K_{M,i}$ is similar for all different synthetases (see Table S4). We use the 80% charging level to estimate this ratio in the simulations (we use the charging capacity of $\xi = V_{max,i}/K_{M,i} = 5/s$). Note that this ratio is proportional to the aminoacylation efficiency $k_{cat,i}/K_{M,i}$.

Considering wobble base pairing via the wobble factor $w(j)$ for each codon j, we reduce the hopping rates of the codons which use the G-U wobble by 39% compared to their G-C wobble counterparts, and those of codons using the I-G wobble by 36% relative to their I-U counterparts, based on the results in (14). In addition to these choices, a 60% reduction has been introduced for the so-called 'missing tRNAs' which are non-perfect matches and which do not have a supplement (15). All wobble examples are listed together with the initial hopping rates in Table S3 for the downscaled cell.

The hopping rate $k(j)$ for each codon j is proportional to the number T_j^c of charged tRNAs for this type of codon, with the proportionality factor r. Considering the wobble base pairing, the hopping rate becomes $k(j) = rT_j^c w(j)$. In order to determine the proportionality factor r, we use the fact that the average hopping rate has been estimated to be $\langle k(j)\rangle = 10/s$ (6)(16)(17). Starting with the assumption of a homogeneous charging level of 80% in the cell, the number of charged tRNAs is estimated as $T_j^c = 0.8\, T_j^t = 0.8\, Tt\, GCN(j)$. Then, the average hopping rate over all codons is given by $\langle k(j)\rangle = r0.8Tt \sum w\,(j)GCN\,(j)/N$ where the sum is over all codons $j = 1, \dots, N$. For a given system size, we can now retrieve the factor of proportionality r , and for the considered downscaled system we obtain $r = 3.696 \times 10^{-3}$. Note that although the average value does not depend on the system size and does not change throughout the simulation, the hopping rate in every single Gillespie step depends on the number of charged tRNAs of the corresponding type.

Furthermore, we use the drop-off rate $\gamma = 5.6 \times 10^{-3}$/s as estimated (18) and the termination rate $\beta = 38.81$/s (19).

The average codon length of mRNA for each GO-Slim class was calculated. The ratio between total number of codons within this category and the number of mRNAs defines the average length of the single representative mRNA of this category. Then we determined the average number of each codon on the representative mRNA by dividing the number of codons of that type by the number of mRNAs within this category. Note that doing so, we neglected codons due to rounding effects, but the loss is less than 0.5%.

The code for the Global Translation Model is available through https://www.ebi.ac.uk/biomodels/ (23) with the following submission identifier following submission identifier: MODEL2001080004. The code is written in C language and adapts some of the structures developed in (22) to access the codons of the mRNA lattices.

Synthetase Sequestration Model

The synthetase sequestration model includes four main transitions for each amino acid type *i*: the binding rate b_i, the charging rate $\tilde{X}_i$, the usage rate $\tilde{k}_i$, and the synthetase-tRNA-complex dissociation rate b_0. We start at time $t = 0$ with 20 amino acids, with a starting configuration where all tRNAs are *empty*, i.e., the number of empty tRNAs is $T_i^e = T_i^t$ and the number of bound and charged tRNAs is zero. To ensure steady state results, we allow the transient time $t_{trans} = 5 \times 10^8\ steps$ to pass before we start to record the number of *charged*, *bound* and *empty* tRNAs of each amino acid type. The average charging level $\langle c_i \rangle$ is equal to the number of charged tRNAs of type *i*, weighted by the time the individual tRNAs of type *i* stay in the charged state, divided by the total number of tRNAs of this type and the total run time. Likewise, we obtain the bound level $\langle b_i \rangle$ and the empty level $\langle e_i \rangle$.

The synthetase sequestration simulation is carried out for the whole cell, i.e., we use the values from Table S4 for the number $E_{0,i}$ of synthetase molecules and the catalytic rate $k_{cat,i}$. The number of synthetase molecules reflects the total amount of synthetase in the cell, i.e, the free synthetase molecules and the ones captured within the synthetase-tRNA-complexes. However, the binding rates depend on the number of free synthetase molecules, i.e, $E_i = \tilde{E}_{0,i} - T_i^b$. Note that it is the total amount which is affected by doxycycline for the glutamine case: $\tilde{E}_{0,Gln} = dE_{0,Gln}$ with the Gln4 protein ratio d.

The code for the Synthetase Sequestration Model is available through the BioModels database https://www.ebi.ac.uk/biomodels/ (23) with the following submission identifier: MODEL2001080005

Specification of parameters; Synthetase Sequestration Model

The usage rate constant $k_3 = 0.1/s$ is estimated to represent the average translation rate. The charging rate constants $k_{2,i} = ak_{cat,i}$ are fitted to comply an average charging level of 80%, which leads to the factor of proportionality of $a = 0.2$. The $k_{cat,i}$ and the enzymatic concentrations of the synthetases $E_{0,i}$ are chosen analogously to the global translation model (Table S4). Note that as a matter of consistency, we adjust the number of glutamine synthetase molecules to the construct, $E_{0,Gln} \approx 10^5$.

The rate constants $k_0 = 43/s$ and $k_{1,Gln} = 0.007/s$ are taken from Uter (20). Note that we have converted the units of the binding rate constant. Using the quasi equilibrium approximation $K_{M,i} = k_0/k_{1,i}$, the binding rate constants of the other amino acids are derived by the relation $V_{max,i}/K_{M,i} = E_{0,i}k_{cat,i}/\left(k_0/k_{1,i}\right)$. Corresponding to the global translation model, the Michaelis-Menten constant $K_{M,i} \propto V_{max,i}$ is proportional to the charging velocity $V_{max,i} = E_{0,i}k_{cat,i}$ with the factor of proportionality $\xi = 15$, which is thought to be the same for all tRNAs, and estimated by using $K_{M,Gln} = 5414$ molecules per cell, corresponding to the value given in Uter (20). Note that in the stochastic simulation we reach a steady state in the bound level, since we never run out of substrate, as the tRNAs are replenished throughout the cycle. Additionally, the dissociation rate constant $k_0 \gg k_{2,i}$ is much larger than the charging rate constants, and therefore, the conditions are fulfilled for the approximation to be appropriate.

General expression for the usage rate

For each tRNA the $usagerate = \sum M_u \, n_{u,i} J_u$ is proportional to the ribosomal current J_u along the mRNA of type u, the number M_u of copies of mRNA of type u, the number $n_{u,i}$ of codons on the mRNA of type u decoded by tRNA of type i.

Every time a ribosome hops with rate k_j from one codon to the next, a charged tRNA is used. Together with the ribosomal density ρ_j, the usage rate can be expressed by

$usagerate = \sum M_u \sum k_{p(j),u} \, \rho_{p(j),u}\left(1 - \rho_{p(j)+1,u}\right)$ with the position $p_{(j),u}$ of the jth codon which is decoded by tRNA i on mRNA of type u.

Autogenous Feedback

For the Global Translation Model (GTM) autogenous feedback we analysed different effects that reduced growth rate has on the initiation rate. In the GTM, the initiation rate is given by $\alpha = \alpha_0 R_{free}$, where R_{free} denotes the number of free ribosomes in the cytoplasm (not engaged in translation), and

α_0 is a proportionality constant that comprises other effects on the initiation, such as the availability of initiation factors or secondary structures on the 5' UTR. In principle, in response to stress we expect that both α_0 and R_{free} are reduced (α_0 decreases because the number of initiation factors can be assumed to decrease with the growth rate, and R_{free} decreases because ribosomes are coupled to the growth rate). Given the experimental data on how the number of ribosomes decreases with Gln4p (Fig. 6), we tested what are the effects of the reduction of α_0 and R_{free} on the simulation results.

Using the data in Figure 6 for calibration of the ribosomal reduction, we obtain the simulation results shown in Fig. S5: when comparing these results to the ones obtained with no feedback (blue solid line), we see that the effect of ribosome reduction is very minor. However, the reduction in the initiation factors availability and therefore α_0 (assumed to decrease proportionally to the growth rate) has a marked effect (shown as a solid red line in Fig. S5), especially on the charging level of the Glutamine codons. Therefore, for the sake of simplicity, we have now only considered the effect of the reduction in α_0.

Moreover, we analysed the effects of tRNA reduction with decreasing growth rate, since in principle not only ribosomes but also tRNAs are downregulated under stress, e.g., through the TOR pathway (21). In order to assess the effect of downregulation of tRNA on our results, we have tested the effects of a tRNA feedback in the GTM, by reducing the amount of tRNA proportionally to the experimentally obtained reduction in ribosomal content. The results are shown in Fig. S5. The green dashed line shows the results of the model incorporating the tRNA feedback. By comparing these results with the ones obtained with the GTM with no feedback (blue solid line), we see that the reduction of the number of tRNAs under stress has only a very minor effect. Therefore, we have neglected it for the sake of simplicity and do not consider tRNA reduction in the rest of the model simulations.

Correlation analysis of the current and the glutamine content

To address the question whether the mRNAs with a high glutamine codon density are more sensitive to increased doxycycline concentration, we did a correlation analysis of the ratio $J_a(+doxy)/J_a(-doxy)$ of the current with and without doxycycline of the GO-Slim category a with the glutamine codon content per mRNA within this GO-Slim category.

In the GTM with no feedback, there is a strong correlation between $J_a(+doxy)/J_a(-doxy)$ and the CAG codon content (Spearman's correlation coefficient of -0.88). Depletion of charged Gln tRNAs causes elongation arrests, and therefore, a reduction in the current.

With autogenous feedback, on the other hand, there is a strong reduction in the correlation with the glutamine content (Spearman's correlation coefficient of 0.22), which is not surprising, because here, the balance between supply and demand is restored; translation initiation rate is decreased so that the charged Gln tRNA available can keep up with the demand.

Note that there is a weaker correlation between reduction in current and the non-rare CAA codon content per mRNA (Spearman's correlation coefficients -0.76 without feedback and 0.18 with feedback).

Figure S3 (panel A) shows the correlation between the CAG content of a mRNA within GO-Slim category a and the ratio $J_a(+doxy)/J_a(-doxy)$ with (red line) and without (blue line) feedback. In panel B, the ratio $J_a(+doxy)/J_a(-doxy)$ with (red line) and without (blue line) feedback together with the CAG content (green line) of a mRNA within GO Slim category a is shown for each GO-Slim category a, ranked by the doxycycline influence on the ratio $J_a(+doxy)/J_a(-doxy)$ without feedback.

Synthetase sequestration at different charging levels

As a matter of consistency with the literature, we used the average 80% tRNA charging level as data to parameterise our model. If we instead use 60% tRNA charging level, as suggested by the experimental results in Fig. 7, the results remain qualitatively the same as shown in Fig. S1A.

The glutamine charging level stays more or less constant whereas the empty level increases with increasing doxycycline concentration (decreasing Gln4 protein ratio) at the expense of the bound level.

Note that a smaller global charging level of the cell leads to a smaller global current. Therefore, the response on doxycycline evolves slightly differently. We use the GTM to inform the SSM how the threshold for the doxycycline sensitive response of the usage rate is shifted; for 80% charging the feedback for the SSM is apparent at Gln4 protein ratio 0.2 which corresponds to the threshold current $J_{thresh} = 0.03/s$ (see Fig. S1B). For 60% charging, however, this value of the current is already reached at the Gln4 protein ratio of 0.35, as shown by the red arrows in Fig. S1B.

Density profiles

Figure S1 shows the profile of the ribosomal density along an mRNA from GO Slim category 66, 'regulation of transport' (bottom), which has a large abundance of slow codons (19).

Comparison to the density profile shown in the main part of the manuscript (Fig. 5H) reveals a similar behaviour, although the two categories are quite different: category 16 has 299 mRNAs in the

downscaled cell with an average length of 158 codons and on average 0.03 CAG codons on each mRNA. In contrast, category 66 has only two mRNAs in the downscaled cell, but with an average length of 920 codons per mRNA and 8.6 CAG codons on each mRNA, see Table S10, below.

category	16	66
**CAA**	33403	1514
**CAG**	258	412
**glutamines**	33661	1926
**all codons**	942334	43881
**mRNAs**	5946	48
**CAA/all codons [%]**	3.5	3.5
**CAG/all codons [%]**	0.03	0.9
**CAA/ glutamines [%]**	99.2	78.6
**CAG/ glutamines [%]**	0.8	21.4
**CAA/ mRNAs**	5.62	31.5
**CAG/ mRNAs**	0.04	8.6
**glutamines/mRNAs**	5.66	40.1

Table S10: A list of number of, CAG, CAA and CAG+CAA (glutamine) codons, number of all codons and mRNAs from the GO-Slim categories 16 and 66. The proportion of the CAG, CAA and CAG+CAA codons compared to all codons in the corresponding category, proportion of the CAG and CAA codons with respect to all glutamine codons within the category and the average number of CAG, CAA and CAG+CAA codons on an mRNA within the category.

The position of the glutamine codons on the mRNA in (Fig. S4) is highlighted by the grey dashed bars. The black line represents the situation without doxycycline. Both, the blue and the red lines represent the situation in the presence of doxycycline, corresponding to a Gln4 protein ratio of 5%, with (red line) and without (blue line) feedback loop.

Without feedback and in the presence of doxycycline, the ribosome density profile in (Fig. S4) is highly inhomogeneous (blue line); ribosomal queues build up behind glutamine codons. When the feedback mechanism is switched on, the profile is much smoother and the queues vanish (red dashed line).

In category 16, the ribosome density along the mRNA in the case with doxycycline is large compared to the case without doxycycline (see figure 6H), whereas in category 66, with doxycycline the

ribosomal density is also large at the entrance of the lattice but decreases rapidly along the mRNA, due to the bottleneck created by the accumulation of rare codons.

In contrast, when the autogenous feedback mechanism is activated, ribosomes are equally distributed in a low density-like regime all over the mRNA, no queueing is visible for any of the two categories. Note that with feedback, the average density in both categories decreases considerably in the presence of doxycycline, which is also reflected in the protein production rate, see (Fig. 6D) in the main text.

Note that the ribosomal particles in the simulation have a footprint of $W = 9$, which leads to the plateau peaks obtained in the case without feedback. Note further that here, the translational site of the ribosome is on the left side, which produces the overhang peaks.

Figures

Figure S1: Panel A: Resulting plot of the steady state charging levels of the glutamine tRNAs as a function of the Gln4 protein ratio (an average of 60% tRNA charging level was used to parameterise our model). Yellow line: mean level $\langle c \rangle$ of charged glutamine tRNAs; Red line: mean level $\langle b \rangle$ of bound glutamine tRNAs; Blue line: mean level $\langle e \rangle$ of empty glutamine tRNAs. The grey line indicates the feedback threshold d = 0.35: for Gln4 protein ratios smaller than 0.35 the usage rate decreases proportionally to the growth rate.

Panel B: Global current J with autogenous feedback of the GTM for two different global charging levels: solid blue line corresponds to the global current for the global charging level at 80% as in Fig.6D of the main text, the dashed blue line corresponds to the global charging level of 60%. The red arrows are a guide for the eye to show how the shift in the feedback threshold for the usage rate in the SSM is obtained.

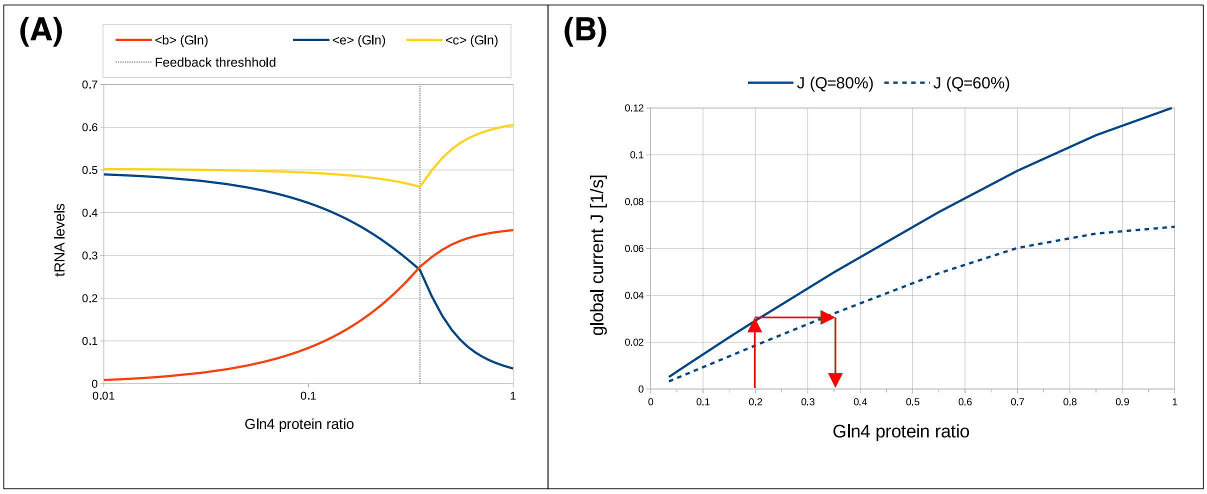

Figure S2; **Depletion of the *KRS1* lysyl-tRNA synthetase using tet-off regulation causes translational induction of GCN4**

The effect of *KRS1* tRNA synthetase shut-off, using doxycycline, on uncharged tRNA accumulation and thus *GCN4* activation was measured using the *GCN4*-lacZ reporter plasmids in a tetO-*KRS1* strain. Reporter gene expression was measured in three independent biological replicates; error bars represent ± 1 standard error of the mean, n=4. Plasmid p180 measures a *GCN4* response, relative to the negative (p226) and positive (p227) controls.

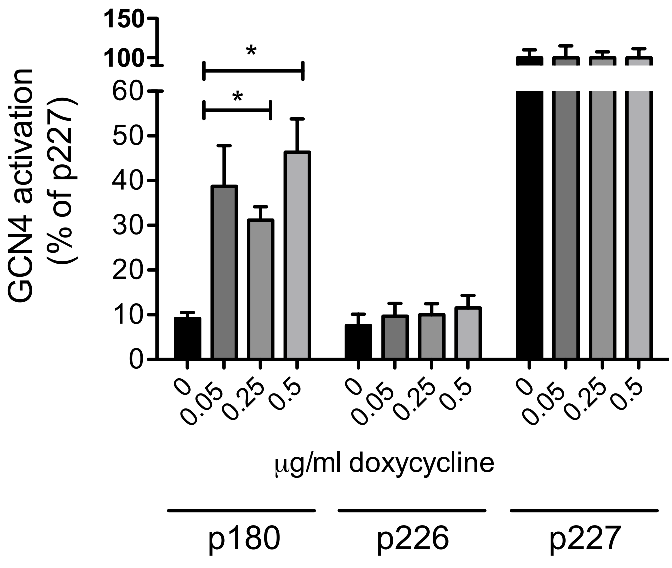

***Figure S3*:** **Phosphorylation of eIF2α in a Gln4 tRNA synthetase tet-off yeast strain in response to doxycycline.** Western blots of total cell lysates were probed with either [panel A] an anti-phospho-eIF2α antibody (phospho-Sui2p [34.7 kDa]; panel A), or a control anti-phosphoglycerate kinase (Pgk1p [44.7 kDa]; panel B) antibody to normalise for lane loading. Lanes 1-3 contain negative control samples, 3-fold overloaded to confirm the absence of phospho-eIF2α (Sui2p); wild-type yeast (lane 1); *GLN4* tet-off Δ*gcn2* (lane 2); *GLN4* tet-off Δ*gcn2*, doxycycline-treated (lane 3). Lanes 4-7 contain *GLN4* tet-off grown in the presence of increasing doxycycline concentrations. Band intensity of phospho-Sui2p was quantified using ImageJ (https://imageJ.nih.gov/ij/index.html), employing Pgk1p to normalise for lane loading variation (panel C).

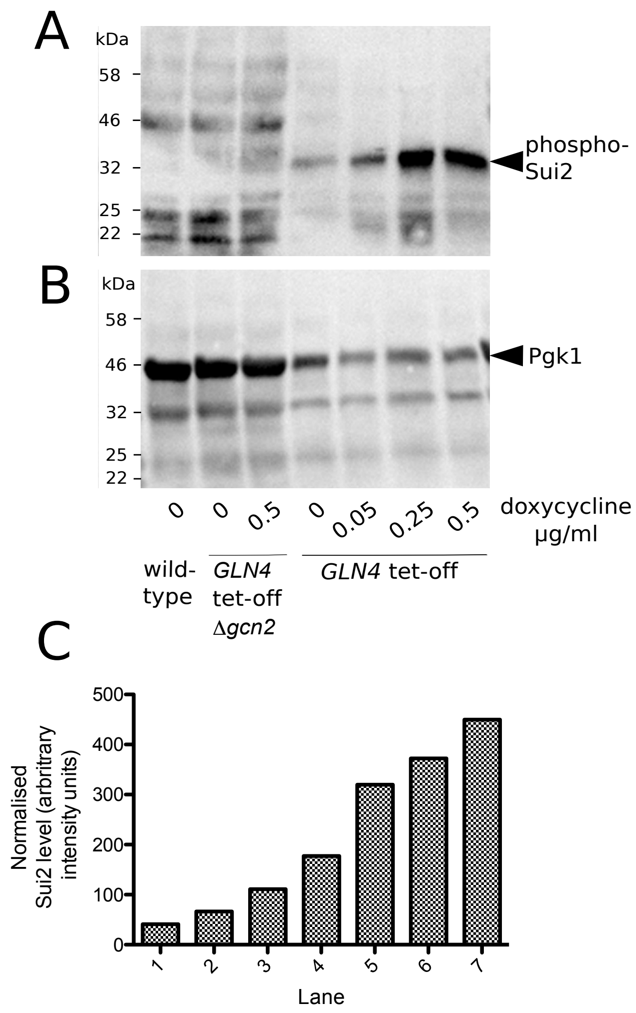

***Figure S4*: Ribosomal density profile along a representative mRNA from GO Slim category 66**

The 'regulation of transport', linear axes (top) and semi-logarithmic axes (bottom) are shown. The black line represents the situation without doxycycline and the blue and red lines represent the situation in the presence of doxycycline, without (blue line) and with (red, dashed line) feedback loop. The positions of the glutamine codons on each mRNA are indicated by the grey, dashed bars.

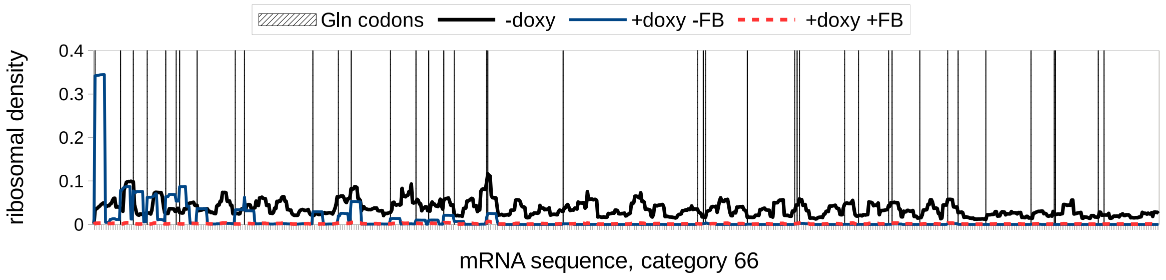

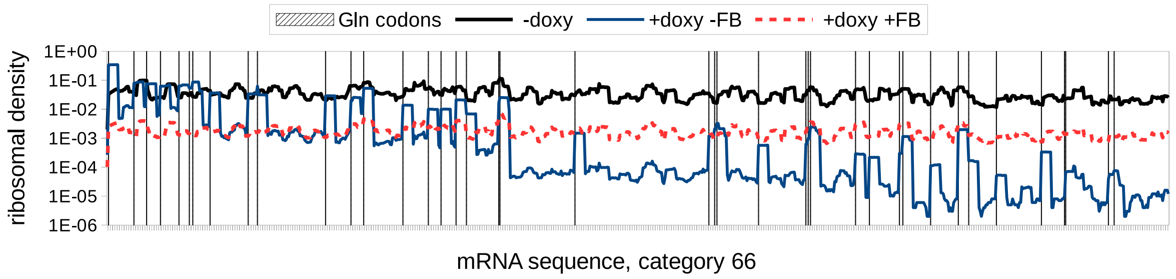

Figure S5: **Different aspects of the autogenous feedback**

No feedback (blue lines) as reference, feedback on the number of ribosomes (green lines), feedback on the number of tRNAs (green dashed lines) and on the initiation factors (red lines). Upper panel: The global current (left) and the global charging level (right) without glutamine tRNAs as functions of the Gln4 protein ratio. Lower panel: The mean charging level of the glutamine tRNAs, CUG (left) and UUG (right). All results have been simulated using a cell size of 100 mRNAs.

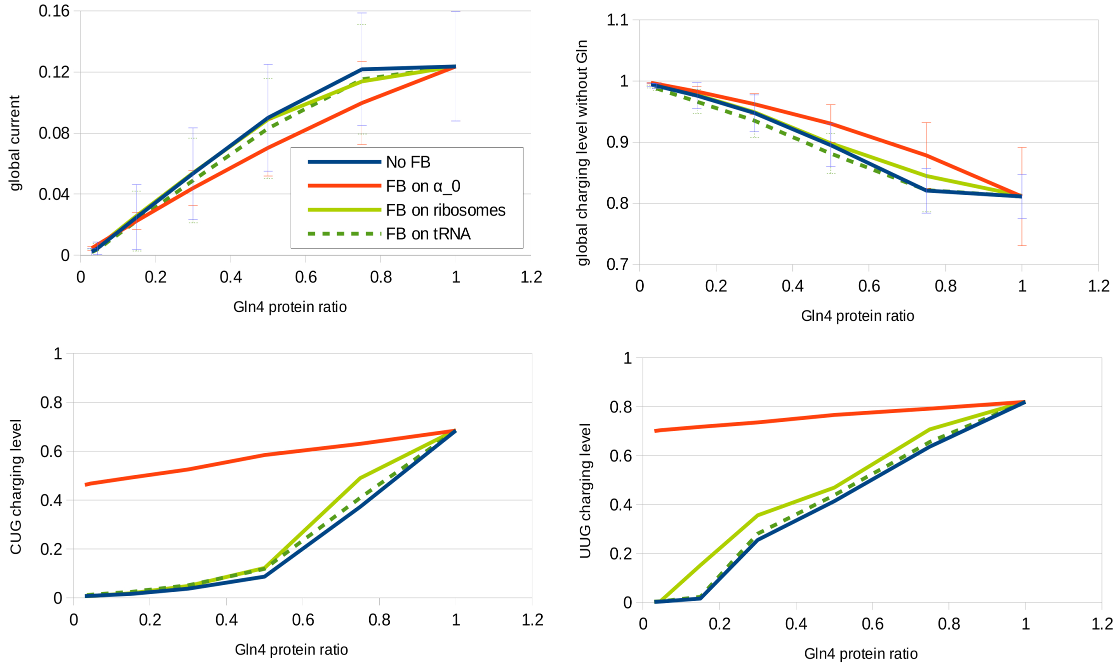

Figure S6: Panel A: correlation between the CAG content of a mRNA within GO Slim category a and the ratio $J_a\,(+doxy)/J_a\,(-doxy)$ with (red line) and without (blue line) feedback. Panel B: the ratio $J_a\,(+doxy)/J_a\,(-doxy)$ with (red line) and without (blue line) feedback together with the CAG content (green line) of a mRNA within GO Slim category a is shown for each GO-Slim category a, ranked by the doxycycline influence on the ratio without feedback.

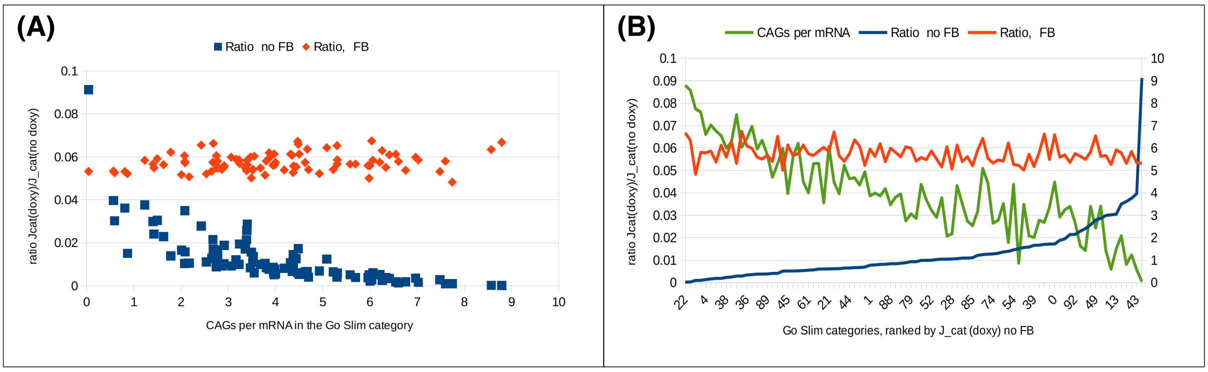

Figure S7; **Hygromycin resistance of a wild-type strain and the Gln tRNA synthetase tet-off strain.** *Panel A-C:* the resistance to hygromycin of the *GLN4* tet-off strain was compared to its progenitor wild-type strain BGY2 by measuring mid-log phase growth rates in cells growing in growing in YPD medium containing doxycycline at either 0, 0.04 and 0.08 μg/ml. *Panel D:*, this data was processed for each concentration of doxycycline to show the growth rates as a percentage of those obtained in 0 μg/ml hygromycin. Cells were grown at 30°C, 400 rpm, in 96-well plates in a LabTech International Omega plate reader. Expressing mid-log phase growth rates in the presence, or absence of hygromycin indicated that depletion of the Gln4p tRNA synthetase did not render the cells more sensitive to hygromycin relative to the effect of hygromycin on wild-type cells (panel D).

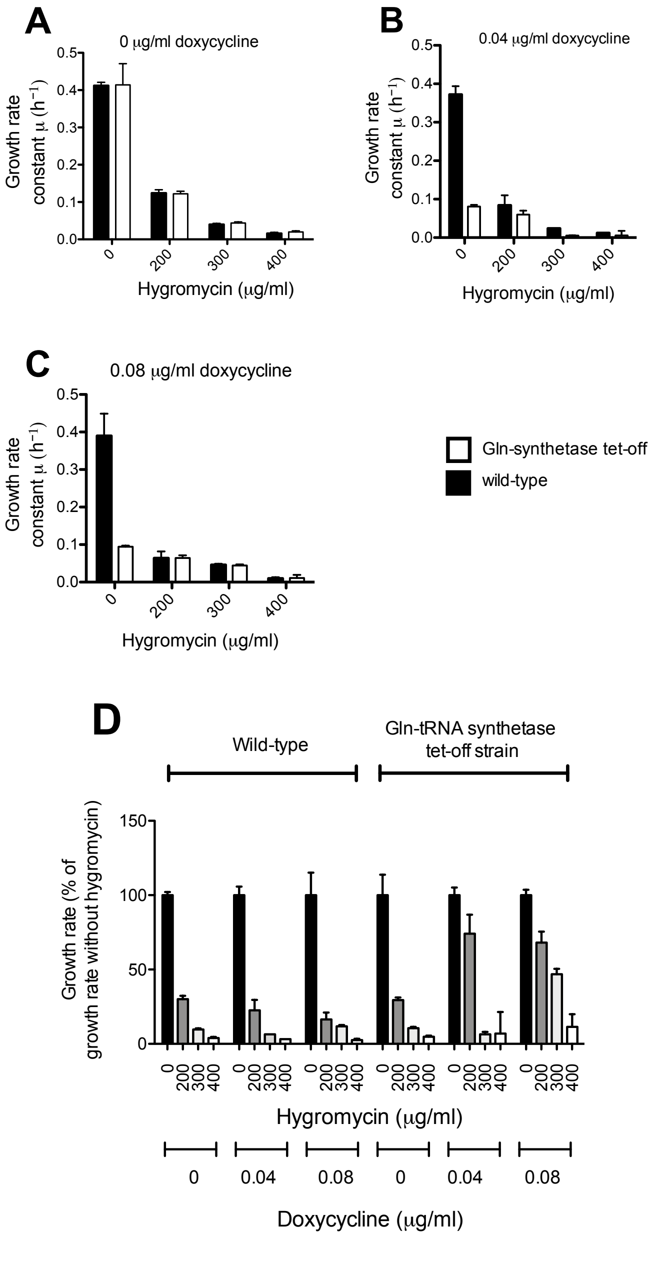

TABLES

Table S1: **Oligonucleotides used in this study**

ID	Name	Sequence 5'->3'
A1	ptetO GLN4 F	AGGGATTTGATGCTTGTTTTAATGAGAAAAAAATATCAGGAGTATCAGCTGAAGCTT CGTACGC
A2	ptetO GLN4 R	AGGGATTTGATGCTTGTTTTAATGAGAAAAAAATATCAGGAGTATCAGCTGAAGCTT CGTACGC
A3	tet GLN4 HA R	CCCAACCTGTGAAAACAGCTGAGTCAATTCTTCTACAGAAGAAGCATAATCAGGAAC ATCGTATGGATACATATAGGCCACTAGTGGATCTG
A4	pET-GLN4 3'	GTTAGCAGCCGGATCTCACTTGGAAGTTGCGTCCTTC
A5	pET GLN4 F	ACGACGACAAGCATATGTATCCATACGATGTTCCTGATTATGCTTCTTCTGTAGAAGA ATTGACTC
A6	$tRNA_{UUG}$ probe (Gln)	TTGTCCGGATCAAAACC
A7	$tRNA_{CUU}$ probe (Lys)	CCCCTAACCTTATGATTAAGAGTC
A8*	Plasmid repair DNA: *GCN2* deletion	GAAGTGAAAGTTGGTGCGCATGTTTCGGCGTTCGAAACTTCTCCGCAGTGAAAGAT AAATCAAATATCAGTTAATCTTTGGTTTTAGAGCTAGAAATAGCAAGTTAAAATAAG GCTAGTCCGTTATCAACTTGAAAAAGTGGCACCGAGTCGG
A9	Genome repair DNA: *GCN2* deletion	GTCTTTTAAGATTTTATAAGCATTGATTTTTTTTTTCAATAATTTTCCGTTCCCCTTAAC ACATACTATGTATAAGCCTTTAAAGATCTGTGCGGCGCTATAACGCATCAGTTAAAG GTAAAGTATATACAATGTCTTAGATACTATATT
A10	gcn2D F	GGAGGAAGCAGTCACCCATC
A11	gcn2D R	GCCTCACACAACATACGCAC
A12	GFP genome integration forward	GCTGACTACGATGCTTTGGACATTGCTAACAGAATCGGTTACATTATGTCTAAAGGT GAAGAATTATTC
A13	GFP genome integration reverse	CATGTTATGAAAGATTTATTGTATATTTAAAGAAAAATTTAAAAATAATATTAAATTT ATTAATTAAATTATTTGTACAATTCATCCATAC
A14	GFP integration gRNA	GAAGTGAAAGTTGGTGCGCATGTTTCGGCGTTCGAAACTTCTCCGCAGTGAAAGAT AAATGATC TCGGTTACATTTAATCTAAT GTTTTAGAGCTAG AAATAGCAAGTTAAAATAAGGCTAGTCCGTTATCAACTTGAAAAAGTGGCACCGAG TCGG
A15	*KRS1* tet-off regulation: forward primer	TTACATACATTGATTTATTGCTCTGTCCTTCCTCCGAGAAATCCGCAGCTGAAGCTTC GTACGC
A16	*KRS1* tet-off regulation: reverse primer	GTTAGCAACACCTTCAGCGGCGGCTTTGACATTATCTTGTTGAGAAGCATAATCAGG AACATCGTATGGATACATATAGGCCACTAGTGGATCTG
A17	$tRNA_{UCU}$ probe (Arg)	CACTCACGATGGGGGTCGAACCCATAATCTTCTGATTAGAAGTCAGACGCGTTGCCA TTA CGCCACGCGAGC
A18	GFP cloning forward	GTTAGCAGCCGGATCCTTATTTGTACAATTCATCCATACCA
A19	GFP cloning reverse	CATATGCTCGAGGATATGTCTAAAGGTGAAGAATTATTCA

*Red text – Indicates *GCN2* gRNA, Green text – Indicates 5' sgRNA

***Table S2:* Parameters used for our downscaled system**

Number of mRNAs, number of codons and the undisturbed initiation rates per GO-Slim category a. In total, there are $n_c = 500124$ and $n_m = 1516$ mRNAs in the downscaled cell fraction and the average initiation rate amounts to $\langle \alpha \rangle = 0.15/s$.

a (GO-Slim)	Number of mRNAs	Number of codons	Av. Length of 1 mRNA	Initiation rate α_a [1/s]
0	2	1182	591	0.0851
1	75	25650	342	0.21
2	23	10718	466	0.21
3	4	2156	539	0.191
4	2	1052	526	0.151
5	-	-	588	-
6	21	8736	416	0.144
7	51	24633	483	0.166
8	23	9085	395	0.107
9	6	1752	292	0.156
10	10	3360	336	0.188
11	17	6086	358	0.18
12	3	1401	467	0.128
13	27	10773	399	0.195
14	3	1533	511	0.119
15	2	1014	507	0.113
16	299	47242	158	0.207
17	7	3360	480	0.124
18	4	1316	329	0.132
19	7	2492	356	0.162
20	3	1458	486	0.11
21	6	2640	440	0.11
22	4	2948	737	0.121
23	6	2460	410	0.101
24	1	494	494	0.13
25	20	7640	382	0.2
26	13	5759	443	0.112
27	3	948	316	0.206
28	4	1492	373	0.177
29	26	11258	433	0.129
30	36	16272	452	0.126
31	5	1970	394	0.15
32	7	2534	362	0.177

a (GO-Slim)	Number of mRNAs	Number of codons	Av. Length of 1 mRNA	Initiation rate α_a [1/s]
33	4	1724	431	0.161
34	10	2080	208	0.191
35	25	7400	296	0.171
36	7	3591	513	0.117
37	20	8920	446	0.201
38	5	2255	451	0.115
39	31	11315	365	0.18
40	70	25200	360	0.187
41	2	1108	554	0.128
42	-	-	834	-
43	35	7630	218	0.207
44	8	3800	475	0.138
45	3	1260	420	0.143
46	29	12557	433	0.133
47	11	3212	292	0.174
48	2	780	390	0.117
49	10	2740	274	0.182
50	15	6270	418	0.152
51	1	454	454	0.0879
52	20	8780	439	0.188
53	8	3352	419	0.132
54	2	818	409	0.102
55	4	1520	380	0.142
56	6	2412	402	0.207
57	5	2585	517	0.0828
58	19	7847	413	0.134
59	22	8470	385	0.133
60	2	1094	547	0.0839
61	6	2370	395	0.179
62	2	1016	508	0.103
63	6	2736	456	0.15
64	2	864	432	0.114
65	15	5280	352	0.189
66	2	1840	920	0.0935
67	37	12432	336	0.171
68	4	2132	533	0.128
69	4	1768	442	0.166
70	19	4275	225	0.209
71	3	1428	476	0.13

a (GO-Slim)	Number of mRNAs	Number of codons	Av. Length of 1 mRNA	Initiation rate α_a [1/s]
72	119	26537	223	0.209
73	6	2874	479	0.119
74	8	2840	355	0.146
75	4	1748	437	0.112
76	58	13166	227	0.201
77	12	5964	497	0.133
78	2	814	407	0.123
79	5	1550	310	0.213
80	4	1928	482	0.158
81	4	1564	391	0.123
82	17	6851	403	0.134
83	3	1077	359	0.138
84	23	12650	550	0.19
85	11	4246	386	0.152
86	23	9844	428	0.122
87	-	-	364	-
88	6	3840	640	0.132
89	4	1728	432	0.14
90	4	1884	471	0.16
91	4	1300	325	0.129
92	3	990	330	0.124
93				
**Sum**	**1516**	**500124**	**-**	**-**
**Average**	**-**	**-**	**430**	**0.15**

***Table S3:* List of amino acids (aa) with corresponding tRNAs (index i) and codons (index j).**

Gene copy number for each tRNA, wobble base pairing for each codon and the maximal hopping rates $k_{max}(j)$ for the downscaled cell. Note that, in general, the hopping rate depends on the number of charged tRNAs, the values given in this table correspond to all tRNAs are 100% charged. Note further, that the wobble factor $w(i)$ used in the equations in the text is given by $w(i) = (1 - wobble)$.

**aa**	**tRNA index i**	**tRNA**	**codon index j**	**codon**	GCN	**wobble**	**$k_{max}(j)$**
Ala	1	IGC	1	GCU	11	0	21.39
Ala	1	IGC	2	GCC	11	0.36	13.69
Ala	2	UGC	3	GCG	6	0.6	4.67
Ala	2	UGC	4	GCA	6	0	11.66
Arg	3	ICG	5	CGU	7	0	13.61
Arg	3	ICG	6	CGC	7	0.36	8.71
Arg	3	ICG	7	CGA	7	0.36	8.71
Arg	4	CCG	8	CGG	1	0	1.94
Arg	5	CCU	9	AGG	1	0	1.94
Arg	6	UCU	10	AGA	12	0	23.33
Asn	7	GUU	11	AAU	11	0.39	13.04
Asn	7	GUU	12	AAC	11	0	21.39
Asp	8	GUC	13	GAU	16	0.39	18.97
Asp	8	GUC	14	GAC	16	0	31.11
Cys	9	GCA	15	UGU	4	0.39	4.74
Cys	9	GCA	16	UGC	4	0	7.78
Gln	10	CUG	17	CAG	1	0	1.94
Gln	11	UUG	18	CAA	9	0	17.50
Glu	12	CUC	19	GAG	2	0	3.89
Glu	13	UUC	20	GAA	15	0	29.16
Gly	14	GCC	21	GGU	16	0.39	18.97
Gly	14	GCC	22	GGC	16	0	31.11
Gly	15	CCC	23	GGG	2	0	3.89
Gly	16	UCC	24	GGA	3	0	5.83
His	17	GUG	25	CAU	8	0.39	9.49
His	17	GUG	26	CAC	8	0	15.55
Ile	18	AAU	27	AUU	13	0	25.27
Ile	18	AAU	28	AUC	13	0.36	16.17
Ile	19	UAU	29	AUA	2	0	3.89
Leu	20	GAG	30	CUU	1	0.39	1.19
Leu	20	GAG	31	CUC	1	0	1.94

aa	tRNA index i	tRNA	codon index j	codon	GCN	wobble	$k_{max}(j)$
Leu	21	UAG	32	CUG	3	0.6	2.33
Leu	21	UAG	33	CUA	3	0	5.83
Leu	22	CAA	34	UUG	10	0	19.44
Leu	23	UAA	35	UUA	7	0	13.61
Lys	24	CUU	36	AAG	14	0	27.22
Lys	25	UUU	37	AAA	8	0	15.55
Met	26	CAU	38	AUG	11	0	21.39
Phe	27	GAA	39	UUU	11	0.39	13.04
Phe	27	GAA	40	UUC	11	0	21.39
Pro	28	IGG	41	CCC	2	0.36	2.49
Pro	28	IGG	42	CCU	2	0	3.89
Pro	29	UGG	43	CCG	10	0.6	7.78
Pro	29	UGG	44	CCA	10	0	19.44
Ser	30	IGA	45	UCU	11	0	21.39
Ser	30	IGA	46	UCC	11	0.36	13.69
Ser	31	CGA	47	UCG	1	0	1.94
Ser	32	UGA	48	UCA	4	0	7.78
Ser	33	GCU	49	AGU	2	0.39	2.37
Ser	33	GCU	50	AGC	2	0	3.89
Thr	34	IGU	51	ACU	11	0	21.39
Thr	34	IGU	52	ACC	11	0.36	13.69
Thr	35	CGU	53	ACG	1	0	1.94
Thr	36	UGU	54	ACA	5	0	9.72
Trp	37	CCA	55	UGG	6	0	11.66
Tyr	38	GUA	56	UAU	8	0.39	9.49
Tyr	38	GUA	57	UAC	8	0	15.55
Val	39	IAC	58	GUU	14	0	27.22
Val	39	IAC	59	GUC	14	0.36	17.42
Val	40	CAC	60	GUG	2	0	3.89
Val	41	UAC	61	GUA	3	0	5.83

Table S4: **Parameters used for the simulation**

Calculation of the catalytic rate k_{cat} for each amino acid type from the estimated number of amino acids added/cell/ second and the number of synthetase/cell. The last column shows the number of tRNA copies/amino acid type.

aa	amino acids added/cell/s	synthetase/cell	kcat (reactions/s)	tRNA copies
Ala	190467	22920	8.31	17
Arg	104064	17540	5.93	21
Asn	117783	5950	19.80	11
Asp	144229	17660	8.17	16
Cys	24576	12970	1.89	4
Gln	87125	25830	3.37	10
Glu	171562	46100	3.72	17
Gly	157047	51950	3.02	21
His	48063	16730	2.87	8
Ile	152007	19790	7.68	16
Leu	211848	80830	2.62	21
Lys	183511	26230	7.00	22
Met	49471	50790	0.97	11
Phe	97812	10270	9.52	11
Pro	102638	13480	7.61	12
Ser	176762	18440	9.59	18
Thr	141140	30520	4.62	17
Trp	23667	11250	2.10	6
Tyr	75072	9500	7.90	8
Val	164360	6750	24.35	19

Table S5; **Gene ontologies significantly enriched in, and common to, gene sets upregulated in response to Gln4 depletion and 3-AT treatment**

***p* values**			
***GLN4*-depletion:**	**3AT treatment**	**Gene ontology class**	**Description**
4.0586E-22	4.786E-26	GO:0008652	cellular amino acid biosynthetic process
7.5122E-20	4.3473E-25	GO:0016053	organic acid biosynthetic process
7.5122E-20	4.3473E-25	GO:0046394	carboxylic acid biosynthetic process
1.0886E-21	5.2633E-25	GO:1901607	alpha-amino acid biosynthetic process
9.2335E-21	6.3954E-23	GO:1901605	alpha-amino acid metabolic process
1.7127E-17	4.1551E-20	GO:0044283	small molecule biosynthetic process
2.0423E-16	4.1081E-19	GO:0006520	cellular amino acid metabolic process
2.7156E-16	4.2686E-19	GO:0019752	carboxylic acid metabolic process
1.3871E-15	1.3062E-18	GO:0043436	oxoacid metabolic process
1.6251E-15	1.5912E-18	GO:0006082	organic acid metabolic process
1.2274E-11	5.454E-17	GO:0044281	small molecule metabolic process
3.667E-10	1.2398E-15	GO:0009067	aspartate family amino acid biosynthetic process
8.1829E-11	9.9498E-13	GO:0009066	aspartate family amino acid metabolic process
3.4377E-09	1.2241E-10	GO:0009086	methionine biosynthetic process
4.3125E-09	3.3538E-10	GO:0006526	arginine biosynthetic process
8.1479E-09	5.7122E-10	GO:0006555	methionine metabolic process
4.5527E-08	6.6122E-10	GO:0000096	sulfur amino acid metabolic process
2.7031E-05	8.1427E-10	GO:0055114	oxidation-reduction process
1.5637E-08	8.2756E-10	GO:0000097	sulfur amino acid biosynthetic process
2.3565E-10	4.2276E-09	GO:0006790	sulfur compound metabolic process
1.2359E-07	2.145E-08	GO:0016829	lyase activity
1.4968E-05	5.3124E-08	GO:0016491	oxidoreductase activity
3.049E-11	8.1241E-08	GO:0044272	sulfur compound biosynthetic process
2.0438E-05	3.8434E-07	GO:0048037	cofactor binding
4.9575E-05	4.6508E-07	GO:0008483	transaminase activity
4.9575E-05	4.6508E-07	GO:0016769	transferase activity, transferring nitrogenous groups
1.4196E-09	3.0789E-06	GO:0006525	arginine metabolic process
2.3477E-05	5.5375E-06	GO:0044282	small molecule catabolic process
1.8657E-08	1.4377E-05	GO:0009084	glutamine family amino acid biosynthetic process
1.2642E-07	2.2184E-05	GO:1901565	organonitrogen compound catabolic process
1.2189E-08	3.2749E-05	GO:0009064	glutamine family amino acid metabolic process

***Table S6;* Gene ontologies significantly enriched in, and common to, gene sets downregulated in response to Gln4 depletion and 3-AT treatment**

***p* values**			
***GLN4*-depletion:**	**3AT treatment**	**Gene ontology class**	**Description**
1.5662E-15	8.347E-92	GO:0043228	non-membrane-bounded organelle
1.5662E-15	8.347E-92	GO:0043232	intracellular non-membrane-bounded organelle
1.2258E-16	4.1402E-91	GO:0030529	intracellular ribonucleoprotein complex
1.2258E-16	4.1402E-91	GO:1990904	ribonucleoprotein complex
1.767E-53	4.8838E-64	GO:0002181	cytoplasmic translation
9.9036E-40	9.3358E-55	GO:0005840	ribosome
1.1195E-46	1.5979E-54	GO:0044445	cytosolic part
1.8043E-26	4.8827E-52	GO:0006412	translation
4.7545E-26	3.1621E-51	GO:0043043	peptide biosynthetic process
1.3219E-22	4.5489E-47	GO:0043604	amide biosynthetic process
1.7481E-23	2.6637E-46	GO:0006518	peptide metabolic process
4.7194E-37	2.2651E-45	GO:0044391	ribosomal subunit
2.7668E-37	2.1065E-43	GO:0003735	structural constituent of ribosome
1.1541E-19	6.86E-40	GO:0043603	cellular amide metabolic process
4.1786E-05	4.526E-36	GO:0000462	maturation of SSU-rRNA from tricistronic rRNA transcript (SSU-rRNA, 5.8S rRNA, LSU-rRNA)
7.866E-31	6.5514E-33	GO:0022627	cytosolic small ribosomal subunit
4.7415E-27	6.8294E-32	GO:0022625	cytosolic large ribosomal subunit
9.7592E-26	3.8026E-31	GO:0005622	intracellular
1.2174E-23	6.4895E-30	GO:1901566	organonitrogen compound biosynthetic process
3.3777E-28	1.1283E-26	GO:0005198	structural molecule activity
7.1011E-24	1.4982E-25	GO:1901564	organonitrogen compound metabolic process
7.1097E-18	1.5934E-25	GO:0015934	large ribosomal subunit
4.5621E-20	2.7434E-20	GO:0015935	small ribosomal subunit
4.7639E-11	4.9283E-19	GO:0044271	cellular nitrogen compound biosynthetic process
3.6033E-10	4.0626E-16	GO:0005737	cytoplasm
2.4835E-10	3.0074E-14	GO:0006407	rRNA export from nucleus
2.4835E-10	3.0074E-14	GO:0051029	rRNA transport
8.0048E-06	5.3341E-12	GO:0044267	cellular protein metabolic process
3.0854E-09	3.2521E-09	GO:0000786	nucleosome
5.9599E-10	1.673E-08	GO:0000788	nuclear nucleosome
5.8362E-07	1.3338E-07	GO:0044815	DNA packaging complex

Table S7; **Gene ontologies significantly enriched in, and common to, proteins upregulated in response to Gln4 depletion (SILAC analysis) and genes upregulated by 3-AT treatment (transcript profile analysis)**

***p* values**			
***GLN4*-depletion:**	**3AT treatment**	**Gene ontology class**	**Description**
1.2379E-28	4.786E-26	GO:0008652	cellular amino acid biosynthetic process
1.4123E-29	4.3473E-25	GO:0016053	organic acid biosynthetic process
1.4123E-29	4.3473E-25	GO:0046394	carboxylic acid biosynthetic process
2.91E-26	5.2633E-25	GO:1901607	alpha-amino acid biosynthetic process
1.4848E-27	6.3954E-23	GO:1901605	alpha-amino acid metabolic process
7.2333E-30	4.1551E-20	GO:0044283	small molecule biosynthetic process
1.6504E-28	4.1081E-19	GO:0006520	cellular amino acid metabolic process
1.2902E-35	4.2686E-19	GO:0019752	carboxylic acid metabolic process
2.2993E-35	1.3062E-18	GO:0043436	oxoacid metabolic process
2.7255E-35	1.5912E-18	GO:0006082	organic acid metabolic process
1.9364E-44	5.454E-17	GO:0044281	small molecule metabolic process
1.1372E-10	1.2398E-15	GO:0009067	aspartate family amino acid biosynthetic process
5.5567E-10	9.9498E-13	GO:0009066	aspartate family amino acid metabolic process
6.8429E-08	4.9081E-11	GO:0009085	lysine biosynthetic process
2.0743E-05	1.2241E-10	GO:0009086	methionine biosynthetic process
3.9311E-09	3.0807E-10	GO:0006553	lysine metabolic process
5.2225E-09	3.3538E-10	GO:0006526	arginine biosynthetic process
5.1918E-06	7.1395E-10	GO:0006081	cellular aldehyde metabolic process
3.8622E-05	8.2756E-10	GO:0000097	sulfur amino acid biosynthetic process
2.6898E-07	2.6861E-08	GO:0019878	lysine biosynthetic process via aminoadipic acid
1.8848E-40	4.5317E-08	GO:0003824	catalytic activity
3.3076E-07	4.6508E-07	GO:0008483	transaminase activity
3.3076E-07	4.6508E-07	GO:0016769	transferase activity, transferring nitrogenous groups
6.9018E-07	3.0789E-06	GO:0006525	arginine metabolic process
2.7726E-06	3.2472E-06	GO:0030170	pyridoxal phosphate binding
6.3369E-14	5.5375E-06	GO:0044282	small molecule catabolic process
1.9819E-06	1.4377E-05	GO:0009084	glutamine family amino acid biosynthetic process
1.2904E-09	3.2749E-05	GO:0009064	glutamine family amino acid metabolic process
1.2379E-28	4.786E-26	GO:0008652	cellular amino acid biosynthetic process
1.4123E-29	4.3473E-25	GO:0016053	organic acid biosynthetic process
1.4123E-29	4.3473E-25	GO:0046394	carboxylic acid biosynthetic process

Table S8: **Proteins identified through SILAC as reduced in concentration in response to Gln tRNA synthetase shut-off (at 1.4-fold or greater reduction)**

Systematic gene name	Gene	Description	Log fold ratio:
YGR159C	NSR1	Nucleolar protein that binds nuclear localization sequences; required for pre-rRNA processing	-0.5073126
YKL180W	RPL17A	Ribosomal Protein of the Large subunit	-0.5087329
YGL028C	SCW11	Soluble Cell Wall protein	-0.5091384
YPL211W	NIP7	Nuclear ImPort	-0.5105569
YGR285C	ZUO1	ZUOtin	-0.5120751
YLR354C	TAL1	TransALdolase	-0.5151069
YGR085C	RPL11B	Ribosomal Protein of the Large subunit	-0.5180315
YNL220W	ADE12	ADEnine requiring	-0.5214528
YGL189C	RPS26A	Ribosomal Protein of the Small subunit	-0.526169
YJL080C	SCP160	S. cerevisiae protein involved in the Control of Ploidy	-0.5308698
YJL136C	RPS21B	Ribosomal Protein of the Small subunit	-0.5385382
YGL234W	ADE5,7	ADEnine requiring	-0.5430009
YHL015W	RPS20	Ribosomal Protein of the Small subunit	-0.5574836
YBR025C	OLA1	Obg-Like ATPase	-0.5574836
YMR058W	FET3	FErrous Transport	-0.5584636
YCR073W-A	SOL2	Suppressor Of Los1-1	-0.5614972
YDL229W	SSB1	Stress-Seventy subfamily B	-0.5638415
YGL147C	RPL9A	Ribosomal Protein of the Large subunit	-0.5644269
YLR448W	RPL6B	Ribosomal Protein of the Large subunit	-0.5651097
YOL120C	RPL18A	Ribosomal Protein of the Large subunit	-0.5651097
YER043C	SAH1	S-Adenosyl-l-Homocysteine hydrolase	-0.5667665
YNL112W	DBP2	Dead Box Protein	-0.5734715
YNL069C	RPL16B	Ribosomal Protein of the Large subunit	-0.5861162
YGR214W	RPS0A	Ribosomal Protein of the Small subunit	-0.5934972
YHR128W	FUR1	5-FluoroURidine resistant	-0.6005552
YDR399W	HPT1	Hypoxanthine guanine PhosphoribosylTransferase	-0.6039764
YBR191W	RPL21A	Ribosomal Protein of the Large subunit	-0.6047356
YNL096C	RPS7B	Ribosomal Protein of the Small subunit	-0.605684
YDR025W	RPS11A	Ribosomal Protein of the Small subunit	-0.6060631
YGL031C	RPL24A	Ribosomal Protein of the Large subunit	-0.6070107
YBR031W	RPL4A	Ribosomal Protein of the Large subunit	-0.6193661
YPR132W	RPS23B	Ribosomal Protein of the Small subunit	-0.6231177
YJR010W	MET3	METhionine requiring	-0.6291001
YDR012W	RPL4B	Ribosomal Protein of the Large subunit	-0.6315234
YAR071W	PHO11	PHOsphate metabolism	-0.6408985
YNL209W	SSB2	Stress-Seventy subfamily B	-0.6429326
YER036C	ARB1	ATP-binding cassette protein involved in Ribosome Biogenesis	-0.6436715
YOR167C	RPS28A	Ribosomal Protein of the Small subunit	-0.6438562

YER177W	BMH1	Brain Modulosignalin Homolog	-0.647729
YMR116C	ASC1	Absence of growth Suppressor of Cyp1	-0.6493855
YML056C	IMD4	IMP Dehydrogenase	-0.6501212
YLR325C	RPL38	Ribosomal Protein of the Large subunit	-0.6520505
YKL001C	MET14	METhionine requiring	-0.652693
YPL127C	HHO1	Histone H One	-0.6548938
YKR057W	RPS21A	Ribosomal Protein of the Small subunit	-0.6570912
YPL131W	RPL5	Ribosomal Protein of the Large subunit	-0.6931412
YPL105C	SYH1	SmY2 Homolog	-0.7021256
YOL109W	ZEO1	ZEOcin resistance	-0.7077898
YAL003W	EFB1	Elongation Factor Beta	-0.7094671
YFL037W	TUB2	TUBulin	-0.7099082
YOR293W	RPS10A	Ribosomal Protein of the Small subunit	-0.7136958
YGR234W	YHB1	Yeast flavoHemogloBin	-0.7277898
YGL123W	RPS2	Ribosomal Protein of the Small subunit	-0.7376002
YLR432W	IMD3	IMP Dehydrogenase	-0.7382923
YJR123W	RPS5	Ribosomal Protein of the Small subunit	-0.7416611
YKL216W	URA1	URAcil requiring	-0.7431272
YER102W	RPS8B	Ribosomal Protein of the Small subunit	-0.7438165
YOR133W	EFT1	Elongation Factor Two	-0.7651952
YJR145C	RPS4A	Ribosomal Protein of the Small subunit	-0.7683326
YLR048W	RPS0B	Ribosomal Protein of the Small subunit	-0.784504
YNL208W			-0.7864291
YBR189W	RPS9B	Ribosomal Protein of the Small subunit	-0.7957669
YAL059W	ECM1	ExtraCellular Mutant	-0.7998333
YOR095C	RKI1	Ribose-5-phosphate Ketol-Isomerase	-0.8062828
YOR063W	RPL3	Ribosomal Protein of the Large subunit	-0.8127036
YHL033C	RPL8A	Ribosomal Protein of the Large subunit	-0.8310665
YDR502C	SAM2	S-AdenosylMethionine requiring	-0.8318772
YNL178W	RPS3	Ribosomal Protein of the Small subunit	-0.8366513
YPL061W	ALD6	ALdehyde Dehydrogenase	-0.8396372
YFL045C	SEC53	SECretory	-0.8425364
YKL054C	DEF1	RNAPII DEgradation Factor	-0.8565475
YPL090C	RPS6A	Ribosomal Protein of the Small subunit	-0.8610025
YNL113W	RPC19	RNA Polymerase C	-0.8742855
YJR070C	LIA1	Ligand of eIF5A	-0.8855744
YLR441C	RPS1A	Ribosomal Protein of the Small subunit	-0.8939834
YGR210C			-0.9280481
YPL043W	NOP4	NucleOlar Protein	-0.9486008
YDR099W	BMH2	Brain Modulosignalin Homolog	-0.9661351
YCR084C	TUP1	dTMP-UPtake	-0.9889936
YLR300W	EXG1	EXo-1,3-beta-Glucanase	-1.0554733
YML063W	RPS1B	Ribosomal Protein of the Small subunit	-1.0614306

YPL079W	RPL21B	Ribosomal Protein of the Large subunit	-1.0688084
YJL177W	RPL17B	Ribosomal Protein of the Large subunit	-1.0727918
YLR206W	ENT2	Epsin N-Terminal homology	-1.0994299
YLR342W	FKS1	FK506 Sensitivity	-1.1277653
YLR249W	YEF3	Yeast Elongation Factor	-1.1402551
YOR252W	TMA16	Translation Machinery Associated	-1.1767696
YDR172W	SUP35	SUPpressor	-1.2705887
YBR092C	PHO3	PHOsphate metabolism	-1.2849286
YOR340C	RPA43	RNA Polymerase A	-1.3810064
YHR057C	CPR2	Cyclosporin A-sensitive Proline Rotamase	-1.6014588
YGR148C	RPL24B	Ribosomal Protein of the Large subunit	-1.6188494
YFL007W	BLM10	BLeoMycin resistance	-1.6273734
YHR216W	IMD2	IMP Dehydrogenase	-1.7110543
YDR101C	ARX1	Associated with Ribosomal eXport complex	-1.7300965
YLR074C	BUD20	BUD site selection	-1.7991702
YNL141W	AAH1	Adenine AminoHydrolase	-1.9590282
YJR004C	SAG1	Sexual AGglutination	-2.035448
YML131W			-3.7443764
YOR168W	GLN4	GLutamiNe metabolism	-5.2097653

Table S9: **Transcriptional response of translation initiation factor genes significantly repressed in the GLN4 tet-off strain in response to doxycycline treatment**

Notes (a)*;* Significantly repressed (green shading, bold font) and induced genes (orange shading) are indicated. (b); A false discovery rate (FDR) of 0.05 was indicated the significance or otherwise of an adjusted *q* value (Benjamini-Hochberg).

**Systematic gene name**	**Gene name**	**Initiation factor**	**Log$_2$ mRNA ratio (+ doxycycline/control)**	***q* value**
YOL139C	*CDC33*	eIF4E	**-0.544**	0.000128
YGL049C	*TIF4632*	eIF4G	**-0.303**	0.019159
YGR162W	*TIF4631*	eIF4G	**-0.931**	0.000128
YKR059W	*TIF1*	eIF4A	**-0.932**	0.000128
YJL138C	*TIF2*	eIF4A	**-0.808**	0.000128
YPR163C	*TIF3*	eIF4B	**-1.207**	0.000128
YBR079C	*RPG1*	eIF3a	**-0.732**	0.000128

Systematic gene name	Gene name	Initiation factor	Log_2 mRNA ratio (+ doxycycline/control)	*q* value
YOR361C	*PRT1*	eIF3b	**-0.371**	0.013003
YMR309C	*NIP1*	eIF3c	**-0.321**	0.0315117
YDR429C	*TIF35*	eIF3g	**-1.09737**	0.000128
YMR146C	*TIF34*	eIF3i	**-1.25764**	0.000128
YLR192C	*HCR1*	eIF3j	**-0.503**	0.000128
YMR260C	*TIF11*	eIF1A	**-0.96252**	0.000128
YNL244C	*SUI1*	eIF1	**-0.72058**	0.000128
YJR007w	*SUI2*	eIF2α	0.195772	0.18319
YPL237W	*SUI3*	eIF2β	**-0.35716**	0.025653
YER025W	*GCD11*	eIF2γ	**-0.76006**	0.000128
YKR026c	*GCN3*	eIF2Bα	0.362512	0.003837
YLR291c	*GCD7*	eIF2Bβ	0.179204	0.181697
YOR260w	*GCD1*	eIF2Bγ	**-0.44972**	0.000592
YGR083c	*GCD2*	eIF2Bδ	**-0.28306**	0.045961
YDR211w	*GCD6*	eIF2Bε	**-0.30185**	0.029318
YPR041w	*TIF5*	eIF5	**-0.64725**	0.000128
YAL035w	*FUN12*	eIF5B	**-0.6244**	0.000128

Table S11: **Summary of the gene ontology categories assigned to the categorical number** a **used in the text.**

a	GO-slim category	category
0	amino acid transport	GO:0006865
1	biological process	GO:0008150
2	carbohydrate metabolic process	GO:0005975
3	carbohydrate transport	GO:0008643
4	cell budding	GO:0007114
5	cell morphogenesis	GO:0000902
6	cell wall organization or biogenesis	GO:0071554
7	cellular amino acid metabolic process	GO:0006520
8	cellular ion homeostasis	GO:0006873
9	cellular respiration	GO:0045333
10	cellular response to DNA damage stimulus	GO:0006974
11	chromatin organization	GO:0006325
12	chromosome segregation	GO:0007059
13	cofactor metabolic process	GO:0051186
14	conjugation	GO:0000746
15	cytokinesis	GO:0000910
16	cytoplasmic translation	GO:0002181
17	cytoskeleton organization	GO:0007010
18	DNA recombination	GO:0006310
19	DNA repair	GO:0006281
20	DNA replication	GO:0006260
21	DNA-templated transcription, initiation	GO:0006352
22	DNA-templated transcription, termination	GO:0006353
22	DNA-templated transcription, elongation	GO:0006354
22	endocytosis	GO:0006897
23	endosomal transport	GO:0016197
24	exocytosis	GO:0006887
25	generation of precursor metabolites and energy	GO:0006091
26	Golgi vesicle transport	GO:0048193
27	histone modification	GO:0016570
28	invasive growth in response to glucose limitation	GO:0001403

a	**GO-slim category**	**category**
29	ion transport	GO:0006811
30	lipid metabolic process	GO:0006629
31	lipid transport	GO:0006869
32	meiotic cell cycle	GO:0051321
33	membrane fusion	GO:0061025
34	mitochondrial translation	GO:0032543
35	mitochondrion organization	GO:0007005
36	mitotic cell cycle	GO:0000278
37	monocarboxylic acid metabolic process	GO:0032787
38	mRNA processing	GO:0006397
39	nuclear transport	GO:0051169
40	nucleobase-containing compound transport	GO:0015931
41	nucleobase-containing small molecule metabolic process	GO:0055086
41	nucleus organization	GO:0006997
42	oligosaccharide metabolic process	GO:0009311
43	organelle assembly	GO:0070925
44	organelle fusion	GO:0048284
45	organelle fission	GO:0048285
45	organelle inheritance	GO:0048308
46	other	-
47	peptidyl-amino acid modification	GO:0018193
48	peroxisome organization	GO:0007031
49	protein alkylation and acylation	GO:0008213
50	protein acylation	GO:0043543
50	protein complex biogenesis	GO:0070271
51	protein dephosphorylation	GO:0006470
52	protein folding	GO:0006457
53	protein glycosylation	GO:0006486
54	protein lipidation	GO:0006497
55	protein maturation	GO:0051604
56	protein modification by small protein conjugation or removal	GO:0070647
57	protein phosphorylation	GO:0006468
58	protein targeting	GO:0006605

a	**GO-slim category**	**category**
59	proteolysis involved in cellular protein catabolic process	GO:0051603
60	pseudohyphal growth	GO:0007124
61	regulation of cell cycle	GO:0051726
62	regulation of DNA metabolic process	GO:0051052
63	regulation of organelle organization	GO:0033043
64	regulation of protein modification process	GO:0031399
65	regulation of translation	GO:0006417
66	regulation of transport	GO:0051049
67	response to chemical	GO:0042221
68	response to heat	GO:0009408
69	response to osmotic stress	GO:0006970
70	response to oxidative stress	GO:0006979
71	response to starvation	GO:0042594
72	ribosomal subunit export from nucleus	GO:0000054
73	ribosome assembly	GO:0042255
73	ribosomal large subunit biogenesis	GO:0042273
73	ribosomal small subunit biogenesis	GO:0042274
73	RNA catabolic process	GO:0006401
74	RNA modification	GO:0009451
75	RNA splicing	GO:0008380
76	rRNA processing	GO:0006364
77	signaling	GO:0023052
78	snoRNA processing	GO:0043144
79	sporulation	GO:0043934
80	telomere organization	GO:0032200
81	transcription from RNA polymerase I promoter	GO:0006360
82	transcription from RNA polymerase II promoter	GO:0006366
83	transcription from RNA polymerase III promoter	GO:0006383
84	translational elongation	GO:0006414
85	translational initiation	GO:0006413
86	transmembrane transport	GO:0055085
87	transposition	GO:0032196
88	tRNA aminoacylation for protein translation	GO:0006418

a	GO-slim category	category
89	tRNA processing	GO:0008033
90	vacuole organization	GO:0007033
91	vesicle organization	GO:0016050
92	vitamin metabolic process	GO:0006766

REFERENCES

1. Gillespie,D.T. (1977) Exact stochastic simulation of coupled chemical reactions. *J. Phys. Chem.*, **81**, 2340–2361.

2. Miura,F., Kawaguchi,N., Yoshida,M., Uematsu,C., Kito,K., Sakaki,Y. and Ito,T. (2008) Absolute quantification of the budding yeast transcriptome by means of competitive PCR between genomic and complementary DNAs. *BMC Genomics*, **9**, 574.

3. Waldron,C. and Lacroute,F. (1975) Effect of growth rate on the amounts of ribosomal and transfer ribonucleic acids in yeast. *J. Bacteriol.*, **122**, 855–865.

4. Warner,J.R. (1999) The economics of ribosome biosynthesis in yeast. *Trends Biochem. Sci.*, **24**, 437–440.

5. Chan,P.P. and Lowe,T.M. (2009) GtRNAdb: a database of transfer RNA genes detected in genomic sequence. *Nucleic Acids Res.*, **37**, D93-7.

6. Arava,Y., Wang,Y., Storey,J.D., Liu,C.L., Brown,P.O. and Herschlag,D. (2003) Genome-wide analysis of mRNA translation profiles in *Saccharomyces cerevisiae*. *Proc. Natl. Acad. Sci. U. S. A.*, **100**, 3889–3894.

7. von der Haar,T. (2008) A quantitative estimation of the global translational activity in logarithmically growing yeast cells. *BMC Syst. Biol.*, **2**, 87.

8. Ghaemmaghami,S., Huh,W.K., Bower,K., Howson,R.W., Belle,A., Dephoure,N., O'Shea,E.K. and Weissman,J.S. (2003) Global analysis of protein expression in yeast. *Nature*, **425**, 737–741.

9. Dittmar,K.A., Sorensen,M.A., Elf,J., Ehrenberg,M. and Pan,T. (2005) Selective charging of tRNA isoacceptors induced by amino-acid starvation. *EMBO Rep.*, **6**, 151–157.

10. Varshney,U., Lee,C.P. and RajBhandary,U.L. (1991) Direct analysis of aminoacylation levels of tRNAs in vivo. Application to studying recognition of *Escherichia coli* initiator tRNA mutants by glutaminyl-tRNA synthetase. *J. Biol. Chem.*, **266**, 24712–24718.

11. Sorensen,M.A. (2001) Charging levels of four tRNA species in *Escherichia coli* Rel(+) and Rel(-) strains during amino acid starvation: a simple model for the effect of ppGpp on translational accuracy. *J. Mol. Biol.*, **307**, 785–798.

12. Jakubowski,H. and Goldman,E. (1984) Quantities of individual aminoacyl-tRNA families and their turnover in *Escherichia coli*. *J. Bacteriol.*, **158**, 769 LP – 776.

13. Kemp,A.J., Betney,R., Ciandrini,L., Schwenger,A.C.M., Romano,M.C. and Stansfield,I. (2013) A yeast tRNA mutant that causes pseudohyphal growth exhibits reduced rates of CAG codon translation. *Mol. Microbiol.*, **87**.

14. Gilchrist,M.A. and Wagner,A. (2006) A model of protein translation including codon bias, nonsense errors, and ribosome recycling. *J. Theor. Biol.*, **239**, 417–434.

15. Bjork,G.R., Huang,B., Persson,O.P. and Bystrom,A.S. (2007) A conserved modified wobble nucleoside (mcm5s2U) in lysyl-tRNA is required for viability in yeast. *RNA*, **13**, 1245–1255.

16. Sorensen,M.A. and Pedersen,S. (1991) Absolute in vivo translation rates of individual codons in *Escherichia coli.* The two glutamic acid codons GAA and GAG are translated with a threefold difference in rate. *J. Mol. Biol.*, **222**, 265–280.

17. Shah,P., Ding,Y., Niemczyk,M., Kudla,G. and Plotkin,J.B. (2013) Rate-limiting steps in yeast protein translation. *Cell*, **153**, 1589–1601.

18. Sin,C., Chiarugi,D. and Valleriani,A. (2016) Quantitative assessment of ribosome drop-off in E. coli. *Nucleic Acids Res.*, **44**, 2528–2537.

19. Ciandrini,L., Stansfield,I. and Romano,M.C. (2013) Ribosome traffic on mRNAs maps to gene ontology: genome-wide quantification of translation initiation rates and polysome size regulation. *PLoS Comput. Biol.*, **9**, e1002866.

20. Uter,N.T., Gruic-Sovulj,I. and Perona,J.J. (2005) Amino Acid-dependent Transfer RNA Affinity in a Class I Aminoacyl-tRNA Synthetase. *J. Biol. Chem.*, **280**, 23966–23977.

21. Huynh,L.-N., Thangavel,M., Chen,T., Cottrell,R., Mitchell,J.M. and Prætorius-Ibba,M. (2010) Linking tRNA localization with activation of nutritional stress responses. *Cell Cycle*, **9**, 3184–3190.

22. W. von Bloh. (2008) Sequential and Parallel Implementation of Networks. In Graben, P., Zhou, C., Thiel, M., and Kurths J., *Lectures in Supercomputational Neuroscience; Understanding Complex Systems*. Springer: Complexity.

23. Chelliah, V. et al. BioModels: ten-year anniversary. Nucl. Acids Res. 2015, 43(Database issue):D542-8 Chelliah,V., Juty,N., Ajmera,I., Ali,R., Dumousseau,M., Glont,M., Hucka,M., Jalowicki,G., Keating,S., Knight-Schrijver,V., Lloret-Villas,A., Natarajan,K.N., Pettit,J,B,, Rodriguez,N., Schubert,M,. Wimalaratne,S.M., Zhao,Y., Hermjakob,H., Le Novère,N. and Laibe,C. (2015) BioModels: ten-year anniversary. *Nucleic Acids Res*., **43** (Database issue):D542-8.
